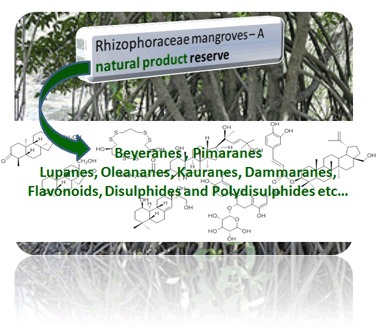# Metabolites and bioactivities of Rhizophoraceae mangroves

**DOI:** 10.1007/s13659-013-0012-0

**Published:** 2013-10-10

**Authors:** Murukesh Nebula, H. S. Harisankar, N. Chandramohanakumar

**Affiliations:** 1 Department of Chemical Oceanography, School of Marine Sciences, Cochin University of Science and Technology, Fine Arts Avenue, Kochi, 682016 Kerala India; 2 Inter University Center for Development of Marine Biotechnology, School of Marine Sciences, Cochin University of Science and Technology, Fine Arts Avenue, Kochi, 682016 Kerala India

**Keywords:** Rhizophoraceae, *Bruguiera*, *Rhizophora*, terpenoids, *Ceriops*

## Abstract

This review examines the chemical compositions and bioactivities of mangrove plants belonging to the Rhizophoraceae family. The Rhizophoraceae family of true mangrove plants is the most common and is also widely distributed species. It consists of 24 species across four genera. Of the 24 species, 12 species remain unexamined for their phytochemical constituents. There have been 268 metabolites reported from 16 species. The key phytochemical constituents identified across the family are the diterpenoids and triterpenoids. The major diterpenoids include pimaranes, beyeranes, kaurenes, dolabranes and labdanes whereas the significant triterpenoids are lupanes, dammaranes and oleananes. Disulphides, dolabranes and labdanes are considered to be the chemotaxonomic markers of the genera *Bruguiera, Ceriops* and *Rhizophora* respectively.

## Introduction

Mangrove plants are potential sources of biologically active chemicals that are discernible from their wide spread application in ethnopharmaceutical practices. There habitat exists under stressful conditions and serve as a bridging ecosystem between freshwater and marine systems. These plants have specially adapted their own morphological structures and physiological mechanisms to their harsh natural surroundings. Pneumatophores, stilt roots and buttresses, with salt-excreting glands found in their leaves, and viviparous propagules are some of the several highly specialized adaptations of this group. The path of photosynthesis in mangroves is different from other glycophytes. Furthermore, there are alterations in other physiological processes such as carbohydrate metabolism or polyphenol synthesis. These plants survive under extreme conditions of salinity, temperature gradients, tidal fluctuations and anoxic soil conditions, with these plants possessing many chemical compounds, which protect them from these destructive elements[1]. Even though extracts from mangroves and mangrove-dependent species possess therapeutic activity against humans, animal and plant pathogens, the specific metabolites responsible for these bioactivities remains to be elucidated.

*To whom correspondence should be addressed. E-mail: nebulamurukesh@gmail.com

## 1 Rhizophoraceae

The global mangrove plant have 84 species belonging to 24 genera and 16 families[2]. Among them, 70 species are true mangroves pertaining to sixteen genera and eleven families whereas fourteen species are semi mangroves belonging to eight genera and five families. According to Wu et. al, suggests the family Rhizophoraceae belongs to true mangrove family, which contains 21 species in four genera[2]. In contrast three more species; *Rhizophora annamalayana*[3], *Kandelia obovata*[4] and *Ceriops zeppeliana blume*[5] become 24 species in four genera in the Rhizophoraceae family of true mangroves. Thus the family Rhizophoraceae include: *Bruguiera* which contains seven species, *Ceriops* (five species), *Kandelia* (two species) and *Rhizophora* (ten species). The distribution of species in Rhizophoraceae family is detailed in Table 1. 54 studies can achieve the validity of ethnomedicines as well as apply the use of mangrove plants in the development of new drugs.

In this review, the compounds identified from this family were listed, and their reported biological activities were compiled. Also chemotaxonamy and importance of further phytochemical research is discussed.

## 2 Chemical Constituents

**2.1** ***Bruguiera*:** The genus *Bruguiera* has six species and one hybrid species which are derived from *B. sexangula*, including *B. cylindrica*, *B. exarista*, *B. gymnorrhiza*, *B. hainessi*, *B. parviflora*, *B. sexangula* and *B. sexangula* var.

**Table 1. Mangroves of Rhizophoraceae family of true mangroves**

| *Bruguiera* | *Ceriops* | *Kandelia* | *Rhizophora* |
|---|---|---|---|
| *B. cylindrica* | *C. decandra* | *K. candel* | *R. apiculata* |
| *B. exarista* | *C. tagal* | *K. obovata* | *R. harrisoni* |
| *B. gymnorrhiza* | *C. tagal* var. *australasica* | | *R. lamarckii* |
| *B. hainessi* | *C. tagal* var. *typical* | | *R. mangle* |
| *B. parviflora* | *C. zeppeliana blume* | | *R. mucronata* |
| *B. sexangula* | | | *R. racemosa* |
| *B. sexangula* var. *rhynchopetala* | | | *R. samoesis* |
| | | | *R. selala* |
| | | | *R. stylosa* |
| | | | *R. annamalayana* |

*rhynchopetala*. The metabolic pattern of this genus has been extensively characterised by a suite of diterpenes and triterpenes. In addition, these species also produce flavonoids, tropane derivatives and cyclic polysulphides. These include 22 metabolites from *B. cylindrica*, 54 metabolites from *B. gymnorrhiza*, nine metabolites from *B. exaristata*, six metabolites from *B. parviflora*, two metabolites from *B. sexangula* and 40 metabolites from *B. sexangula* var *rhynchopetala* were identified so far. A total of 114 metabolites have been reported form this genus including. A detailed list of chemical compounds identified from *Bruguiera* is recorded in Table 2.

Three sulphur compounds along with an alkaloid brugine were reported from the stem and bark of *B. cylindrica* by Japanese scientists of during 1975–1976. 20 years later, a number of oleananes and lupanes (triterpenoids) and one kaurane (diterpenoid) were reported. The first report on the chemical constituents of *B. gymnorrhiza* dates back to 1978 in which Sarkar and Ganguly reported a new triterpenoid called gymnorhizol (3-epi-$\delta$-amyrin). Since then various triterpenoids (lupanes, oleananes, ursanes, dammaranes) and diterpenoids (kauranes, pimaranes, beyaranes) along with sulphur compounds, sterols and aromatic compounds were reported from this plant.

Only two reports are available regarding the chemical constituents of *B. exaristata*. As part of their investigation on tumor inhibitory plants, in 1969 Loder and Russell identified the presence of alkaloids (brugine, tropine and tropine esters of acetic, iosbutyric, iosvaleric, propionic, n-butyric and benzoic acids) in the bark extracts of *B. exaristata*[6] while a pronounced accumulation of 1-d-1-*O*-methyl-muco-inositiol in the young leaves of *B. exaristata* was reported later by Richter and his team[7].

In a continuing search for bioactive constituents from Thai medicinal plants, Chumkaew and his team isolated and elucidated a new triterpenoid ester 3-(*Z*)-caffeoyllupeol along with five other tritepenoids; lupeol caffeate, 3-(*Z*)-coumaroyllupeol, dioslupecin A, lupeol and lupenone from the fruits of *B. Parviflora*[8]. The earliest work regarding the chemical constituents of mangroves deals with the isolation and characterization of the tropine 1,2-dithiolane-3-carbonylate named as bruguine from the stem bark of *B. sexangula*[9]. Later the same team identified additional alkaloids from the same plant as part of their investigation on tumor inhibitory plants[6]. In a study focusing on the marine fauna and flora from Chinese coasts, Li and his coworkers collected samples of the mangrove *B. sexangula* from Hainan Province, China. On separation of an EtOAc-soluble fraction of a methanol extract of the title plant, they isolated a new triterpene, named sexangulic acid[10].

Investigation of Chinese mangrove plants led to the isolation and characterisation of 13 compounds; three new diterpenes; six known diterpenes, a new dithiobenzoquinone two cyclic disulfides and 2,6-dimethoxy-1,4-benzoquinone from the EtOH extract of the stem of *B. sexangula* var. *rhynchopetala*[11]. Further several triterpenoids and sterols were reported[12]. Recently, a continuous investigation for chemical diversity of *B. sexangula* var. *rhynchopetala* led to the isolation and characterization of six new phenolic constituents named rhyncosides A–F, together with twelve known compounds including two phenolic glycosides, four flavonoids, and six lignan derivatives[13].

**Alkaloids**

**1**

**2** R = H
**3** R = Me
**4** R = Ph
**5** R = $CH(CH_3)_2$
**6** R = $CH_2CH(CH_3)_2$
**7** R = $(CH_2)_2CH_3$
**8** R = $CH_2CH_3$

**Figure 1.** Alkaloids from Rhizophoraceae mangroves

**2.2 *Ceriops***: The genus of *Ceriops* has two species and two varieties, namely *C. tagal* (Perr.), *C. decandra*, *C. tagal* var. *australasica* and *C. tagal* var. typical. These plants are valued for their rich tannin content and are a rich source of pentacyclic triterpenoids[17]. To date, 30 metabolities from *C. decandra* and 72 metabolites from *C. tagal* are known. Thus a total of 92 metabolites including 45 diterpenoids (23 dolabranes, six dimeric diterpenoids, four beyeranes, five kauranes, and seven pimaranes) and 45 tritepenoids (35 lupanes, seven dammaranes, one oleanane, one ursane, and one abietane) along with two steroids have been reported so far from this genus.

On examination of the roots of *C. decandra* collected from the Kauvery estuary (Parangipettai coast), Anjaneyulu and his team isolated and characterised twelve diterpenoids[30,31,32]. Subsequently, two novel triterpene esters were isolated from the leaves of *C. decandra* in addition to 16 known triterpenes[33]

by scientists from Thailand. Dolabranes (diterpenoids) are the marker metabolites of *C. tagal*. These compounds can be used as chemotaxonomic markers of this plant. Dimeric diterpenoids (tetraterpenoids) and triterpenoids of lupane, dammarane, pimarane groups are also found in this plant. One abietane and an oleanane triterpenoid were also isolated from the stems and twigs of *C. tagal*. The chemical constituents identified from *ceriops* are listed in Table 3.

**Table 2. Chemical constituents from the genus *Bruguiera***

| Compound Class and Name | Plant | Plant Part | References |
|---|---|---|---|
| *Alkaloids* | | | |
| brugine (**1**) | *B. cylindrica* | stem and bark | 14 |
| | *B. exaristata* | stem bark | 6 |
| tropine (**2**) | *B. exaristata* | stem bark | 6 |
| | *B. sexangula* | stem bark | 6 |
| tropine acetate (**3**) | *B. exaristata* | stem bark | 6 |
| | *B. sexangula* | stem bark | 6 |
| tropine benzoate (**4**) | *B. exaristata* | stem bark | 6 |
| | *B. sexangula* | stem bark | 6 |
| tropine isobutyrate (**5**) | *B. exaristata* | stem bark | 6 |
| | *B. sexangula* | stem bark | 6 |
| tropine isovalerate (**6**) | *B. exaristata* | stem bark | 6 |
| | *B. sexangula* | stem bark | 6 |
| tropine n-butyrate (**7**) | *B. exaristata* | stem bark | 6 |
| | *B. sexangula* | stem bark | 6 |
| tropine propionate (**8**) | *B. exaristata* | stem bark | 6 |
| | *B. sexangula* | stem bark | 6 |
| *D-Friedooleananes (Triterpenoids)* | | | |
| 3*α*-taraxerol (**9**) | *B. cylindrica* | fruits | 15 |
| 3*α*-*E*-caffeoyltaraxerol (**10**) | *B. cylindrica* | fruits and hypocotyls | 16 |
| 3*α*-*E*-coumaroyltaraxerol (**11**) | *B. cylindrica* | fruits | 15 |
| 3*α*-*E*-feruloyltaraxerol (**12**) | *B. cylindrica* | fruits | 15 |
| 3*α*-*Z*-coumaroyltaraxerol (**13**) | *B. cylindrica* | fruits | 15 |
| 3*α*-*Z*-feruloyltaraxerol (**14**) | *B. cylindrica* | fruits | 15 |
| 3*β*-taraxerol (**26**) | *B. cylindrica* | fruits | 15 |
| 3*β*-*E*-feruloyltaraxerol (**17**) | *B. cylindrica* | fruits | 15 |
| 3*β*-*Z*-feruloyltaraxerol (**23**) | *B. cylindrica* | fruits | 15 |
| taraxerone (**25**) | *B. sexangula var. rhynchopetala* | stem | 12 |
| *Lupanes (Triterpenoids)* | | | |
| 3*α*-lupenol (**32**) | *B. cylindrica* | fruits and hypocotyls | 16 |
| 3*α*-*E*-coumaroyllupeol (**34**) | *B. cylindrica* | fruits and hypocotyls | 16 |
| 3*α*-*Z*-coumaroyllupeol (**37**) | *B. cylindrica* | fruits and hypocotyls | 16 |
| 3*β*-*E*-caffeoyllupeol B (**44**) | *B. cylindrica* | fruits and hypocotyls | 16 |
| 3*β*-*Z*-caffeoyllupeol (**38**) | *B. parviflora* | fruits | 8 |
| 3*β*-*E*-coumaroyllupeol (**45**) | *B. cylindrica* | fruits and hypocotyls | 16 |
| 3*β*-*Z*-coumaroyllupeol (**55**) | *B. cylindrica* | fruits and hypocotyls | 16 |
| | *B. parviflora* | fruits | 8 |
| betulin (**57**) | *B. gymnorrhiza* | leaves | 17 |
| lupenone (**64**) | *B. cylindrica* | fruits and hypocotyls | 16 |
| | *B. sexangula* var. *rhynchopetala* | stem | 12 |
| | *B. parviflora* | fruits | 8 |
| lupeol (**65**) | *B. cylindrica* | fruits and hypocotyls | 16 |
| | *B. gymnorrhiza* | leaves | 17 |
| | *B. sexangula* var. *rhynchopetala* | stem | 12 |
| | *B. parviflora* | fruits | 8 |
| *trans*-hydroxy-cinnamoyl lupeol (**66**) | *B. sexangula* var. *rhynchopetala* | stem | 12 |
| dioslupecin A (**57**) | *B. parviflora* | fruits | 8 |
| *Oleanane (Triterpenoids)* | | | |

| | | | |
|---|---|---|---|
| oleanolic acid (**70**) | *B. gymnorrhiza* | leaves | 17 |
| *β*-amyrin (**71**) | *B. gymnorrhiza* | leaves | 17 |
| *β*-amyril palmitate (**72**) | *B. sexangula* var. *rhynchopetala* | stem | 12 |
| *Ursanes (Triterpenoids)* | | | |
| ursolic acid (**73**) | *B. gymnorrhiza* | leaves | 17 |
| *α*-amyrin (**74**) | *B. gymnorrhiza* | leaves | 17 |
| *Dammaranes (Triterpenoids)* | | | |
| bruguierin **A** (**75**) | *B. gymnorrhiza* | flowers | 18 |
| bruguierin B (**76**) | *B. gymnorrhiza* | flowers | 18 |
| bruguierin C (**77**) | *B. gymnorrhiza* | flowers | 18 |
| *Triterpene alcohol* | | | |
| gymnorhizol (3-*epi*-*δ*-amyrin) (**85**) | *B. gymnorrhiza* | leaves | 19 |
| *Lanostanes (Triterpenoids)* | | | |
| sexangulic acid (**86**) | *B. sexangula* | stem | 10 |
| squalene *(Triterpenoid)* | | | |
| squalene (**87**) | *B. sexangula* var. *rhynchopetala* | stem | 12 |
| *Fatty acids* | | | |
| linoleic acid (**88**) | *B. gymnorrhiza* | leaves | 20 |
| linolenic acid (**89**) | *B. gymnorrhiza* | leaves | 20 |
| palmitic acid (**90**) | *B. gymnorrhiza* | leaves | 20 |
| *Steroids* | | | |
| 3-*O*-*α*-L-rhamnopyranosyl-(+)-catechin-(4*α*→2)phloroglucinol (**91**) | *B. gymnorrhiza* | bark | 21 |
| campesterol (**92**) | *B. gymnorrhiza* | leaves | 17 |
| cholesterol (**93**) | *B. gymnorrhiza* | leaves | 17 |
| daucosterol (**94**) | *B. sexangula* var. *rhynchopetala* | stem | 12 |
| *β*-sitosterol (**96**) | *B. sexangula* var. *rhynchopetala* | stem | 12 |
| | *B. gymnorrhiza* | leaves | 17 |
| stigmaste-7-en-3*β*-ol (**98**) | *B. gymnorrhiza* | leaves | 17 |
| stigmasterol (**99**) | *B. gymnorrhiza* | leaves | 17 |
| *α*-hydroxy-sitosterol (**100**) | *B. sexangula* var. *rhynchopetala* | stem | 12 |
| *Kauranes (Diterpenoids)* | | | |
| (16*R*)-13,17-epoxy-16-hydroxy-*ent*-kaur-9(11)-en-19-al (**101**) | *B. sexangula var. rhynchopetala* | stem | 11 |
| 13,16,17-trihydroxy-*ent*-9(11)-kaurene-19-oic acid (**102**) | *B. gymnorrhiza* | stem | 22 |
| 13-hydroxy-16-ent-kauren-19-al (**103**) | *B. gymnorrhiza* | stem | 22 |
| | *B. gymnorrhiza* | bark | 23 |
| 16,17-dihy-droxy-ent-9(11)-kaurene-19-al (**107**) | *B. gymnorrhiza* | stem | 22 |
| | *B. sexangula* var. *rhynchopetala* | stem | 11 |
| 16,17-dihydroxy-*ent*-9(11)-kauren-19-oic acid (**108**) | *B. gymnorrhiza* | stem | 22 |
| 16,17-dihydroxy-19-nor-*ent*-kaur-9(11)-en-3-one (**109**) | *B. sexangula* var. *rhynchopetala* | stem | 11 |
| 16-*ent*-kaurene-13,19-diol **(115)** | *B. gymnorrhiza* | stem | 22 |
| | *B. gymnorrhiza* | bark | 23 |
| | *B. cylindrica* | roots | 24 |
| 16-*ent*-kauren-19-ol (**110**) | *B. gymnorrhiza* | stem | 22 |
| 16*H*-17,19-*ent*-kauranediol (**104**) | *B. gymnorrhiza* | stem | 22 |
| 16*H*-17-hydroxy-*ent*-kauran-19-oic acid (**105**) | *B. gymnorrhiza* | stem | 22 |
| 16,17-dihydroxy-*ent*-kauran-19-al (**106**) | *B. gymnorrhiza* | stem | 22 |
| 17-chloro-13,16-dihydroxy-*ent*-kauran-19-al (**111**) | *B. gymnorrhiza* | stem | 22 |
| ceriopsin F (**113**) | *B. sexangula* var. *rhynchopetala* | stem | 11 |
| steviol (**120**) | *B. gymnorrhiza* | bark | 23 |

| | | | |
|---|---|---|---|
| methyl(16*R*)-13,17-epoxy-16-hydroxy-*ent*-kaur-9(11)-en-19-oate (**119**) | *B. sexangula* var. *rhynchopetala* | stem | 11 |
| | *B. gymnorrhiza* | bark | 23 |
| methyl-16*α*,17-dihydroxy-*ent*-kaur-9(11)-en-19-oate (**116**) | *B. sexangula* var. *rhynchopetala* | stem | 11 |
| | *B. gymnorrhiza* | stem | 22 |
| methyl-16,17-dihydroxy-*ent*-kauran-19-oate (**117**) | *B. gymnorrhiza* | stem | 22 |
| *Pimaranes (Diterpenoids)* | | | |
| 15(*S*)-isopimar-7-en-15,16-diol (**123**) | *B. gymnorrhiza* | stem | 25 |
| | *B. gymnorrhiza* | root bark | 23 |
| *ent*-8(14)-pimarene-15*R*,16-diol (**128**) | *B. gymnorrhiza* | stem | 25 |
| *ent*-8(14)-pimarene-1*α*,15*R*,16-triol (**129**) | *B. gymnorrhiza* | Stem, root bark | 25, 23 |
| isopimar-7-ene-1β,15R,16-triol **(130)** | *B. gymnorrhiza* | stem | 25 |
| (5*R*,9*S*,10*R*,13*S*,15*S*)*ent*-8(14)-pimarene-1-oxo-15*R*,16-diol (**122**) | *B. gymnorrhiza* | stem | 25 |
| (1αH,15*R*)-ent-pimar-8(14)-ene-1,15,16-triol (**121**) | *B. sexangula* var. *rhynchopetala* | stem | 11 |
| *Beyeranes (Diterpenoids)* | | | |
| (4*R*,5*S*,8*R*,9*R*,10*S*,13*S*)-*ent*-17-hydroxy-16-oxobeyeran-19-al (**135**) | *B. gymnorrhiza* | stem | 22 |
| *ent*-17-hydroxy-16-oxobeyer-9(11)-en-19-al (**136**) | *B. sexangula* var. *rhynchopetala* | stem | 11 |
| *Sulphur compounds* | | | |
| 4-hydroxy-1,2-dithiolane (**144**) | *B. cylindrica* | stem and bark | 14 |
| brugierol (**145**) | *B. cylindrica* | stem and bark | 14 |
| | *B. gymnorrhiza* | flowers, leaves and stem | 26, 27 |
| | *B. sexangula* var. *rhynchopetala* | stem | 11 |
| bruguiesulfurol (**146**) | *B. gymnorrhiza* | Flowers, leaves and stem | 26, 27 |
| *cis*-3,30-dihydroxy-1,5,10,50-tetrathiacyclodecane (**147**) | *B. gymnorrhiza* | leaves and stem | 27 |
| gymnorrhizol (**148**) | *B. gymnorrhiza* | leaves and stem | 27 |
| isobrugierol **(149)** | *B. cylindrica* | stem and bark | 14 |
| | *B. gymnorrhiza* | flowers, leaves and stem | 26, 27 |
| | *B. sexangula var. rhynchopetala* | stem | 11 |
| neogymnorrhizol (**150**) | *B. gymnorrhiza* | leaves and stem | 27 |
| *trans*-3,30-dihydroxy-1,5,10,50-tetrathiacyclodecane (**151**) | *B. gymnorrhiza* | leaves and stem | 27 |
| (–)-3,4-dihydro-3-hydroxy-7-methoxy-2*H*-1,5-benzodithiepine-6,9-dione (**152**) | *B. sexangula* var. *rhynchopetala* | stem | 11 |
| *Aromatic compounds* | | | |
| 1-(3-hydroxyphenyl)-hexane-2,5-diol (**153**) | *B. gymnorrhiza* | stem | 28 |
| 2,3-dimethoxy-5-propylphenol (**154**) | *B. gymnorrhiza* | branch | 29 |
| 3-(3-hydroxybutyl)-1,1-dimethylisochroman-6,8-diol (**155**) | *B. gymnorrhiza* | stem | 28 |
| bruguierol A (**156**) | *B. gymnorrhiza* | stem | 28 |
| bruguierol B (**157**) | *B. gymnorrhiza* | stem | 28 |
| bruguierol C (**158**) | *B. gymnorrhiza* | stem | 28 |
| bruguierol D (**159**) | *B. gymnorrhiza* | branch | 29 |
| *Carbohydrates* | | | |
| 1-d-1-*O*-methyl muco inositiol (**161**) | *B. exaristata* | leaves | 7 |
| *Benzoquinone* | | | |
| 2,6-dimethoxy-1,4-benzoquinone (**162**) | *B. sexangula* var. *rhynchopetala* | stem | 11 |
| *Phenolic glycosides* | | | |

| | | | |
|---|---|---|---|
| 1-[α-L-rhamnopyranosyl-(1→6)-β-D-glucopyranosyloxy]-3,4,5-trimethoxybenzene (**163**) | *B. sexangula* var. *rhynchopetala* | stem | 13 |
| 3,4,5-trimethoxyphenyl-β-D-glucopyranoside (**164**) | *B. sexangula* var. *rhynchopetala* | stem | 13 |
| rhyncoside A (**168**) | *B. sexangula* var. *rhynchopetala* | stem | 13 |
| rhyncoside B (**169**) | *B. sexangula* var. *rhynchopetala* | stem | 13 |
| rhyncoside C (**170**) | *B. sexangula* var. *rhynchopetala* | stem | 13 |
| rhyncoside D (**171**) | *B. sexangula* var. *rhynchopetala* | stem | 13 |
| *Flavonoids* | | | |
| myricetin-3-*O*-rutinoside (**199**) | *B. sexangula* var. *rhynchopetala* | stem | 13 |
| nicotiflorin (**200**) | *B. sexangula* var. *rhynchopetala* | stem | 13 |
| rutin (**205**) | *B. sexangula* var. *rhynchopetala* | stem | 13 |
| tricin (**206**) | *B. sexangula* var. *rhynchopetala* | stem | 13 |
| *Lignans* | | | |
| (+)-5′-methoxyisolariciresinol-9′-β-*D*-xylopyranoside (**207**) | *B. sexangula* var. *rhynchopetala* | stem | 13 |
| (+)-lyoniresinol-3α-*O*-α-L-rhamnopyranoside (**208**) | *B. sexangula* var. *rhynchopetala* | stem | 13 |
| brugunin A (**209**) | *B. gymnorrhiza* | branch | 29 |
| hedyotisols A (**210**) | *B. sexangula* var. *rhynchopetala* | stem | 13 |
| hedyotisols B (**211**) | *B. sexangula* var. *rhynchopetala* | stem | 13 |
| hedyotisols C (**212**) | *B. sexangula* var. *rhynchopetala* | stem | 13 |
| lyoniside (**213**) | *B. sexangula* var. *rhynchopetala* | stem | 13 |
| rhynocoside E (**214**) | *B. sexangula* var. *rhynchopetala* | stem | 13 |
| rhynocoside F (**215**) | *B. sexangula* var. *rhynchopetala* | stem | 13 |

**Table 3. Chemical constituents of *Ceriops***

| Compound Class and Name | Plant | Plant Part | References |
|---|---|---|---|
| *Dolabranes (Diterpenoids)* | | | |
| tagalsin A (**223**) | *Ceriops tagal* | stem and twigs | 34 |
| | | aerial parts | 35 |
| tagalsin B (**224**) | *C. tagal* | stem and twigs | 34 |
| | | aerial parts | 35 |
| tagalsin C (**225**) | *C. tagal* | stem and twigs | 34 |
| | | aerial parts | 35 |
| | | roots | 36 |
| tagalsin D (**226**) | *C. tagal* | stem and twigs | 34 |
| tagalsin E (**227**) | *C. tagal* | stem and twigs | 34 |
| | | aerial parts | 35 |
| tagalsin F (**228**) | *C. tagal* | stem and twigs | 34 |
| | | aerial parts | 35 |
| | | roots | 37, 38 |
| tagalsin G (**229**) | *C. tagal* | stem and twigs | 34 |
| | | aerial parts | 35 |
| tagalsin H (**230**) | *C. tagal* | stem and twigs | 34 |
| tagalsin O (**231**) | *C. tagal* | aerial part | 35 |
| tagalsin P (**232**) | *C. tagal* | stems and twigs | 38 |
| tagalsin Q (**233**) | *C. tagal* | stems and twigs | 38 |
| tagalsin R (**234**) | *C. tagal* | stems and twigs | 38 |
| tagalsin S (**235**) | *C. tagal* | stems and twigs | 38 |
| tagalsin T (**236**) | *C. tagal* | stems and twigs | 38 |
| tagalsin U (**237**) | *C. tagal* | stems and twigs | 38 |
| (5*S**,8*S**,9*S**,10*R**,13*S**)-3-hydroxy-16-nor-2-oxodolabr-3-en-15-oic acid (**219**) | *C. tagal* | stems and twigs | 38 |
| (5*S**,8*S**,9*S**,10*R**,13*S**)-3,16-dihydroxydolabar-3-ene-2,15-dione (**218**) | *C. tagal* | stems and twigs | 38 |

| | | | |
|---|---|---|---|
| (5*S**,8*S**,9*S**,10*,13*S**)-2-hydroxy-16-nor-3-oxodolabr-1,4(18)dien-15-oic acid (**217**) | *C. tagal* | stems and twigs | 38 |
| (5*S**,8*S**,9*S**,1*R**,13*S**)-dolabr-3-ene-15-,16-diol (**220**) | *C. tagal* | stems and twigs | 38 |
| (5*S**,8*S**,9*S**,10*R**,13*S**)-dolabr-4(18)-ene-15,16-diol (**216**) | *C. tagal* | stems and twigs | 38 |
| erythroxydiol Y (**222**) | *C. tagal* | stems and twigs | 38 |
| dolabr-4(17),15(16)-dien-3-one (**238**) | *C. tagal* | roots | 39 |
| 7-glycoloyl-2-hydroxy-1,4b,7,10a-tetramethyl-4a,4b,5,6,7,8,8a,9,10,10a-decahydrohenanthren-3(4*H*)-one (**239**) | *C. tagal* | roots | 40 |
| *Dimeri diterpenoids* | | | |
| tagalsns I (**244**) | *C. tagal* | stems and twigs | 41 |
| | *C. tagal* | roots | 36 |
| tagalsins J (**245**) | *C. tagal* | stems and twigs | 41 |
| tagalsin L (**241**) | *C. tagal* | roots | 42 |
| tagalsin M (**242**) | *C. tagal* | roots | 42 |
| tagalsin N (**243**) | *C. tagal* | roots | 42 |
| 8(14)-enyl-pimar-2'(3')-en-4'(18')-en-15'(16')endolabr-16,15,2',3'-oxoan-16-one (**240**) | *C. tagal* | roots | 36 |
| *Beyranes (Diterpenoids)* | | | |
| ceropsin A (**137**) | *C. tagal* | roots | 30 |
| ceiopsin B (**138**) | *C. tagal* | roots | 30 |
| criopsin G (**139**) | *C. tagal* | roots | 31 |
| isosteviol (**140**) | *C. tagal* | roots | 31 |
| *Auranes (Diterpenoids)* | | | |
| cerioprin E (**112**) | *C. tagal* | roots | 32 |
| ceriopsin F (**113**) | *C. tagal* | roots | 31 |
| steviol (**120**) | *C. tagal* | roots | 31 |
| methyl-ent-16*β*,17-dihydroxy-9(11)-kauren-19-oate (**118**) | *C. tagal* | roots | 31 |
| *ent*-16*β*,17-dihydroxy-9(11)-kauren-19-oic acid (**114**) | *C. decandra* | roots | 31 |
| *Dammarane (Triterpenoids)* | | | |
| cereotagalol A (**78**) | *C. tagal* | hypocotyls and fruits | 43 |
| cereotagalol B (**79**) | *C. tagal* | hypocotyls and fruits | 43 |
| cereotagaloperoxide (**80**) | *C. tagal* | hypocotyls and fruits | 43 |
| dammarenediol II (**81**) | *C. tagal* | hypocotyls and fruits | 43 |
| fouquierol (**82**) | *C. tagal* | hypocotyls and fruits | 43 |
| isofouquierol (**83**) | *C. tagal* | hypocotyls and fruits | 43 |
| ocotillol II (**84**) | *C. tagal* | hypocotyls and fruits | 43 |
| *Oleananes (Triterpenoids)* | | | |
| oleanolic acid (**70**) | *C. tagal* | hypocotyls and fruits | 43 |
| ursane (Triterpenoid) | | | |
| ursolic acid (**73**) | *C. decandra* | leaves | 33 |
| *Lupanes (Triterpenoids)* | | | |
| 28-hydroxylup-20(29)-en-3-one (**27**) | *C. tagal* | aerial parts | 44 |
| | *C. tagal* | roots | 36 |
| 3-*epi*-betulin (**28**) | *C. tagal* | aerial parts | 44 |
| 30-nor-lup-3*β*-ol-2-one (**29**) | *C. decandra* | leaves | 33 |
| 3-*epi*-betulinic acid (**30**) | *C. tagal* | aerial parts | 44 |
| 3-oxo-lup-20(29)-en-28-oic acid (**31**) | *C. tagal* | roots | 36 |
| 3*α*-betulinic acid (**33**) | *C. tagal* | hypocotyls and fruits | 43 |
| | *C. decandra* | leaves | 33 |
| 3*α*-*O*-*trans*-coumaroylbetulinic acid (**35**) | *C. tagal* | aerial parts | 44 |

| | | | |
|---|---|---|---|
| 3α-*O*-*trans*-feruloylbetulinic acid (**36**) | *C. tagal* | aerial parts | 44 |
| 3β-*E*-caffeoyllupeol (**44**) | *C. decandra* | leaves | 33 |
| 3β-*O*-*cis*-coumaroylbetulin (**39**) | *C. tagal* | aerial parts | 44 |
| 3β,20-dihydroxylupane (**40**) | *C. decandra* | leaves | 33 |
| 3β-acetylbetulinic acid (**41**) | *C. tagal* | hypocotyls and fruits | 43 |
| 3β-*E*-caffeoylbetulin (**42**) | *C. tagal* | hypocotyls and fruits | 43 |
| 3β-*E*-caffeoylbetulinic acid (**43**) | *C. tagal* | hypocotyls and fruits | 43 |
| 3β-*E*-coumaroyllupeol (**45**) | *C. decandra* | leaves | 33 |
| | *C. tagal* | hypocotyls and fruits | 43 |
| 3β-*E*-feruloylbetulin (**46**) | *C. decandra* | leaves | 33 |
| | *C. tagal* | hypocotyls and fruits | 43 |
| 3β-*E*-feruloylbetulinic acid (**47**) | *C. tagal* | hypocotyls and fruits | 43 |
| 3β-*E*-feruloyllupeol (**48**) | *C. decandra* | leaves | 33 |
| | *C. tagal* | hypocotyls and fruits | 43 |
| 3β-hydroxylupan-29-oic acid (**49**) | *C. decandra* | leaves | 33 |
| 3β-*O*-*cis*-coumaroylbetulinic acid (**50**) | *C. tagal* | aerial parts | 44 |
| 3β-*O*-*trans*-feruloylbetulin (**51**) | *C. tagal* | aerial parts | 44 |
| 3β-*O*-*cis*-feruloylbetulin (**52**) | *C. tagal* | aerial parts | 44 |
| 3β-*O*-*trans*-coumaroylbetulinic acid (**53**) | *C. tagal* | aerial parts | 44 |
| 3β-*O*-*trans*-coumaroylbetulin (**54**) | *C. tagal* | aerial parts | 44 |
| 3β-*Z*-coumaroyllupeol (**55**) | *C. decandra* | leaves | 33 |
| 3β-*Z*-feruloyllupeol (**56**) | *C. decandra* | leaves | 33 |
| | *C. tagal* | hypocotyls and fruits | 43 |
| betulin (**57**) | *C. decandra* | leaves | 33 |
| | *C. tagal* | hypocotyls and fruits | 43 |
| | *C. tagal* | aerial parts | 44 |
| betulinaldehyde (**58**) | *C. decandra* | leaves | 33 |
| betulinic acid (**59**) | *C. decandra* | leaves | 33 |
| | *C. tagal* | hypocotyls and fruits | 43 |
| betulonic acid (**60**) | *C. tagal* | hypocotyls and fruits | 43 |
| lup-20(29)-en-3β,28-diol (**61**) | *C. tagal* | roots | 39 |
| | *C. tagal* | roots | 36 |
| lup-20(29)-en-3β,30-diol (**62**) | *C. decandra* | leaves | 33 |
| lup-20(29)-en-3β-hydroxy-28-oic (**63**) | *C. tagal* | roots | 39 |
| lupenone (**64**) | *C. decandra* | leaves | 33 |
| lupeol (**65**) | *C. decandra* | leaves | 33 |
| | *C. tagal* | hypocotyls and fruits | 43 |
| | *C. tagal* | aerial parts | 44 |
| | *C. tagal* | roots | 39 |
| *Pimaranes (Diterpenoids)* | | | |
| 8,15-repoxypimaran-16-ol (**125**) | *C. decandra* | roots | 31 |
| ceriopsin C (**126**) | *C. decandra* | roots | 30 |
| ceriopsin D (**127**) | *C. decandra* | roots | 30 |
| *ent*-8(14)-pimarene-15*R*,16-diol (**128**) | *C. tagal* | roots | 45 |
| | *C. tagal* | stems and twigs | 38 |
| isopimar-8(14)-en-15,16-diol (**131**) | *C. tagal* | roots | 39 |
| isopimar-8(14)-en-16-hydroxy-15-one (**132**) | *C. tagal* | roots | 39 |
| methoxy-ent-8(14)-pimarenely-15-one (**133**) | *C. tagal* | roots | 45 |
| *Abietane (Triterpenoid)* | | | |
| abieta-8,11,13-trien-18-oic acid (**246**) | *C. tagal* | stems and twigs | 38 |
| Steroids | | | |
| stigmasterol (**99**) | *C. tagal* | roots | 45 |
| β-sitosterol (**96**) | *C. tagal* | roots | 45 |

**Table 4. Chemical constituents of the bark of *Kandelia candel***

| Compound Class | Compound Name |
|---|---|
| *Propelargonidin dimers* | afzelechin-(4*α*→8)- afzelechin (**180**)<br>afzelechin-(4*α*→8)- catechin (**181**)<br>afzelechin-(4*α*→8)- epicatechin (**182**) |
| *Procyanidin trimers* | epicatechin-(4*β*→6)-epicatechin-(4*β*→6)-epicatechin (**188**)<br>epicatechin-(4*β*→6)-epicatechin-(4*β*→8)-catechin (**189**)<br>epicatechin-(4*β*→6)-epicatechin-(4*β*→8)-epicatechin (**190**) |
| *Proanthocynadins* | cinchonain Ia (**184**)<br>cinchonain Ib (**185**)<br>cinchonain IIa (**186**)<br>cinchonain IIb (**187**)<br>kandelins A-1, A-2, B-1, B-2, B-3, B-4 (**193–198**)<br>proanthicyanidin B-1, B-2, C-1 (**201–203**)<br>proanthicyanidin trimer (**204**) |
| *Flavan-3-ols* | (–)-epicatechin (**172**)<br>(+)-afzeleczhin (**173**)<br>(+)-catechin (**174**)<br>(+)-gallocatechin (**176**) |

**Table 5. Chemical constituents of the genus *Rhizophora***

| Compound Class and Name | Plant | Plant Part | References |
|---|---|---|---|
| *D-Friedooleananes (Triterpenoids)* | | | |
| 3*β*-*O*-*E*-coumaroyl-taraxerol (**15**) | *R. stylosa* | stems and twigs | 48 |
| 3*β*-*E*-caffeoyltaraxerol (**16**) | *R. mucronata* | fruits | 49 |
| 3*β*-*O*-*Z*)coumaroyl-taraxerol (**19**) | *R. stylosa* | stems and twigs | 48 |
| 3*β*-taraxerol acetate (**20**) | *R. stylosa* | stems and twigs | 48 |
| 3*β*-taraxerol formate (**21**) | *R. stylosa* | stems and twigs | 48 |
| 3*β*-*Z*-caffeoyltaraxerol (**22**) | *R. mucronata* | fruits | 49 |
| 3*β*-*Z*-*p*-coumaroyltaraxerol (**24**) | *R. mucronata* | fruits | 49 |
| 3*β*-*E*-*p*-coumaroyltaraxerol (**18**) | *R. mucronata* | fruits | 49 |
| careaborin-(3*β*-*E*-*p*-coumaroyltaraxerol) (**18**) | *R. apiculata* | leaves | 47 |
| | *R. stylosa* | leaves | 50 |
| | *R. stylosa* | leaves | 50 |
| taraxerol (**28**) | *R. apiculata* | leaves | 47 |
| | *R. mangle* | leaves and stems | 51 |
| | *R. stylosa* | leaves | 50 |
| | *R. stylosa* | stems and twigs | 48 |
| | *R. mucronata* | fruits | 49 |
| taraxerone (**26**) | *R. stylosa* | leaves | 50 |
| taraxeryl-*cis*-*p*-hydroxycinnamate (**24**) | *R. apiculata* | leaves | 47 |
| *Lupanes (Triterpenoids)* | | | |
| *trans*-hydroxycinnamoyllupeol (**66**) | *R. mangle* | leaves and stems | 51 |
| lupeol (**65**) | *R. apiculata* | stem | 52 |
| | *R. mucronata* | leaves | 17 |
| | *R. mucronata* | stem bark | 53 |
| *Oleananes (Triterpenoids)* | | | |
| 15*α*-hydroxy-*β*-amyrin (**67**) | *R. stylosa* | stems and twigs | 48 |
| 3*β*-*O*-(*E*)-(4-methoxy)-cinnamoyl-15*α*-hydroxyl-*β*-amyrin (**68**) | *R. mucronata* | stem bark | 53 |
| 3*β*-*O*-(*E*)-coumaroyl-15*α*-hydroxy-*β*-amyrin (**69**) | *R. stylosa* | stems and twigs | 48 |
| oleanolic acid (**70**) | *R. mucronata* | leaves | 17 |
| *β*-amyrin (**71**) | *R. mucronata* | root bark | 54 |
| | *R. mucronata* | leaves | 17 |
| *Ursanes (Triterpenoids)* | | | |

| | | | |
|---|---|---|---|
| ursolic acid (**73**) | *R. mucronata* | leaves | 17 |
| *α*-amyrin (**74**) | *R. mucronata* | root bark | 54 |
| *Aliphatic alcohols* | | | |
| dotriacontanol (**247**) | *R. apiculata* | heartwood | 55 |
| hentriacontanol (**248**) | *R. apiculata* | heartwood | 55 |
| nonacosanol (**249**) | *R. apiculata* | heartwood | 55 |
| octacosanol (**250**) | *R. apiculata* | heartwood | 55 |
| triacontanol (**251**) | *R. apiculata* | heartwood | 55 |
| *Aliphatic saturated carboxylic acids* | | | |
| doicosanoic (**252**) | *R. apiculata* | heartwood | 55 |
| henicosanoic (**253**) | *R. apiculata* | heartwood | 55 |
| hentriacontanoic (**254**) | *R. apiculata* | heartwood | 55 |
| heptacosanoic (**255**) | *R. apiculata* | heartwood | 55 |
| hexatriacontanoic (**256**) | *R. apiculata* | heartwood | 55 |
| octacosanoic (**257**) | *R. apiculata* | heartwood | 55 |
| pentacosanoic (**258**) | *R.a apiculata* | heartwood | 55 |
| tetracosanoic (**259**) | *R. apiculata* | heartwood | 55 |
| tetratriacontanoic (**260**) | *R. apiculata* | heartwood | 55 |
| triacontanoic (**261**) | *R. apiculata* | heartwood | 55 |
| tritriacontanoic (**262**) | *R. apiculata* | heartwood | 55 |
| *Steroids* | | | |
| campesterol (**92**) | *R. apiculata* | heartwood | 55 |
| daucosterol (**94**) | *R. mucronata* | root bark | 54 |
| | *R. stylosa* | leaves | 50 |
| ergosta-7,22-dien-3*β*-ol (**95**) | *R. apiculata* | stem | 52 |
| sitosterol (**96**) | *R. apiculata* | heartwood | 55 |
| | *R. mucronata* | root bark | 54 |
| | *R. stylosa* | leaves | 50 |
| sitosteryl-3-glucoside (**97**) | *R. apiculata* | heartwood | 55 |
| stigmasterol (**99**) | *R. apiculata* | heartwood | 55 |
| *Aromatic compound* | | | |
| syringaldehyde (**160**) | *R. apiculata* | heartwood | 55 |
| *Benzoquinone* | | | |
| 2,6-dimethoxy-*p*-benzoquinone (**162**) | *R. apiculata* | heartwood | 55 |
| *Labdanes(Diterpenoids)* | | | |
| apiculol (**263**) | *R. apiculata* | roots | 56 |
| rhizophorin A (**264**) | *R. mucronata* | roots | 57 |
| | *R. mucronata* | roots | 58 |
| *Beyeranes (Diterpenoids)* | | | |
| rhizophorin B (**141**) | *R. mucronata* | roots | 58 |
| rhizophorin C (**142**) | *R. mucronata* | roots | 58 |
| rhizophorin D (**143**) | *R. mucronata* | roots | 58 |
| *Pimaranes (Diterpenoids)* | | | |
| 15(*S*)-isopimar-7-en-1-oxo-15,16-diol (**124**) | *R. apiculata* | stem | 52 |
| rhizophorin E (**134**) | *R. mucronata* | roots | 58 |
| *Kauranes (Diterpenoids)* | | | |
| 13,16*α*,17-trihydroxy-*ent*-9(11)-kauren-19-oic acid (**102**) | *R. apiculata* | stem | 52 |
| 16*R*-13,17-epoxy-16-hydroxy-ent-kaur-9(11)-en-19-al (**101**) | *R. apiculata* | stem | 52 |
| *ent*-12,17-epoxy-16*β*-hydroxy-9(11)-kauren-19-oate (**112**) | *R. apiculata* | stem | 52 |
| methyl-*ent*-16*β*,17-dihydroxy-9(11)-kauren-19-oate (**118**) | *R. apiculata* | stem | 52 |
| methyl-ent-kaur-9(11)-*ent*-13,17-expoxy-16-hydroxy-19-oate (**119**) | *R. apiculata* | stem | 52 |
| *Sesquiterpene* | | | |

| | | | |
|---|---|---|---|
| mucronatone (**265**) | *R. mucronata* | fruits | 49 |
| *Carbohydrate* | | | |
| 1-d-*O*-methyl-muco-inositol (**161**) | *R. mucronata* | roots | 7 |
| *Hopanoid* | | | |
| adian-5-en-3-ol (**266**) | *R. mucronata* | stem bark | 53 |
| *Phenolic compounds* | | | |
| atranorin (**165**) | *R. mucronata* | root bark | 54 |
| protocatechuic acid (**167**) | *R. stylosa* | leaves | 50 |
| isovanillic acid (**166**) | *R. stylosa* | leaves | 50 |
| *Xanthone(aromatic ketone)* | | | |
| lichixanthone (**267**) | *R. mucronata* | root bark | 54 |
| *Aliphatic ketone* | | | |
| palmitone (**268**) | *R. mucronata* | root bark | 54 |
| *Flavonoids* | | | |
| rutin (**205**) | *R. stylosa* | leaves | 50 |
| astilbin (**183**) | *R. stylosa* | leaves | 50 |
| (–)-3,7-*O*-diacetyl-epicatechin (**178**) | *R. stylosa* | stems and twigs | 59 |
| (–)-epicatechin (**172**) | *R. stylosa* | stems and twigs | 59 |
| | *R. stylosa* | stems | 60 |
| (–)-3-O-acetyl-epicatechin (**179**) | *R. stylosa* | stems and twigs | 59 |
| (–)-3,3′,4′,5,7-O- pentaacetyl-epicatechin (**177**) | *R. stylosa* | stems and twigs | 59 |
| (+)-afzelechin (**173**) | *R. stylosa* | stems and twigs | 59 |
| (+)-catechin (**174**) | *R. stylosa* | stems and twigs | 59 |
| | *R. stylosa* | stems | 60 |
| proanthocyanidin B2 (**202**) | *R. stylosa* | stems and twigs | 59 |
| glabraoside A (**191**) | *R. stylosa* | stems | 60 |
| glabraoside B (**192**) | *R. stylosa* | stems | 60 |
| cinchonain IIa (**186**) | *R. stylosa* | stems | 60 |
| cinchonain IIb (**187**) | *R. stylosa* | stems | 60 |
| (+)-catechin-3-*O*-*α*-L-rhamnoside (**175**) | *R. stylosa* | stems | 60 |
| cinchonain Ia (**186**) | *R. stylosa* | stems | 60 |
| | *R. stylosa* | stems | 60 |
| cinchonain Ib (**185**) | *R. stylosa* | stems and twigs | 59 |
| | *R. stylosa* | stems | 60 |

**2.3 *Kandelia*:** There are two species in the mangrove genus *Kandelia*: *K. candel* and *K. obovata*. Only one report is available regarding the chemical constituents of plants of this genus. A few tannin compounds have been reported from *K. candel*. Investigation of *K. obovata* for its chemical constituents remains to be observed. 24 phenolic compounds including three propelargonidin dimmers, three procyanidin trimers, fourteen proanthocyanidins and four flavan-3-ols have been isolated from the bark of *K. candel Druce*[46].

**2.4 *Rhizhophora*:** The mangrove genus *Rhizophora* has ten species: *R. apiculata*, *R. harrisonii*, *R. lamarckii*, *R. mangle*, *R. mucronata*, *R. racemosa*, *R. samoesis*, *R. selala*, *R. stylosa* and *R. annamalayana*. Of these ten species, chemical constituents have only been reported in *R. apiculata*, *R. mangle*, *R. mucronata*, and *R. stylosa*. These reports reveal a total of 34 metabolites from *R. apiculata*, two metabolites from *R. mangle*, 23 metabolites from *R. mucronata* and 25 metabolites from *R. stylosa*, thus a total of 81 different metabolites from the genera *Rhizophora*, with details shown in Table 5.

The chemical investigation carried out by Majumdar and Patra in 1976 resulted in the isolation of *β*-amyrin, *β*-amyrone, taraxerol, *β*-sitosterol, and triacantanol from *R. apiculata*[47]. Later in early nineties Kokpol and his team had identified three terpenoids, five long chain aliphatic alcohols, eleven long chain aliphatic saturated carboxylic acids, three steroids, 2,6-dimethoxy-*p*-benzoquinone, syringaldehyde and sitosteryl 3-glucoside from this plant species. Also, five kauranes, one labdane and one pimarane diterpenoids and one lupane triterpenoid are reported so far from *R. apiculata*.

The study conducted by Williams et. al[51] reported the isolation and chemical characterisation of taraxerol and cinnamoyllupeol, two triterpenoids from the leaves and stems of *Rhizophora mangle* L. A variety of steroids, diterpenoids and triterpenoids were reported from the leaves and bark of *R. mucronata.* A few beyeranes (diterpenoids) were identified from this plant, and are unique to this species. Only triterpenoids of the classes oleananes and D-friedooleananes from *R. stylosa* are reported to date.

**Figure 2.** D-friedooleananes (triterpenoids) from Rhizophoraceae mangroves

## 3 Bioactivities

**3.1 Bioactivities of Compounds Identified:** With stably transfected HepG2 cells, three new dammarane triterpenes; bruguierins A–C and a new cyclic 4-hydroxy-dithiosulfonate-bruguiesulfurol as well as two known 4-hydroxydithiolane-1-oxides; brugierol and isobrugierol, were isolated from the flowers of *Bruguiera gymnorrhiza*. These phytochemicals activated an antioxidant response element (ARE luciferase activation) with $EC_{50}$ values of 7.8, 9.4, 15.7, 56.7, 3.7 and 1.8 µM, respectively. Furthermore, bruguierin A, brugierol and isobrugierol also inhibited phorbol ester-induced NFκB (nuclear factor-κB) luciferase activation with an $IC_{50}$ value of 1.4, 85.0 and 14.5 µM respectively, while bruguierin A and brugierol selectively inhibited cyclooxygenase-2 (COX-2) activity with an $IC_{50}$ value of 0.37 and 6.1 µM respectively[18,26].

The compounds 16*α*-17,19-*ent*-kauranediol; 13-hydroxy-16-*ent*-kaurene-19-ol and 16-*ent*-kaurene-19-ol showed promising activity against K-562 (human chronic myeloid leukemia) and L-929 (mouse fibroblasts) of which 16-*ent*-kaurene-19-ol showed the greatest selectivity for K-562 ($IC_{50}$ 6.8 µg/mL)[22]. (5*R*,9*S*,10*R*,13*S*,15*S*)*ent*-8(14)-pimarene-1-oxo-15,16-diol showed moderate cytotoxic activities against L-929[25].

The 15 membered macrocyclic polysulfide, gymnorrhizol, possesses an novel carbon skeleton which was isolated from *B. gymnorrhiza* and exhibited potent inhibitory activity against protein tyrosine phosphatase 1B (PTP1B). PTP1B is an enzyme involved in the regulation of insulin signaling and which is regarded as a key for treatment of type III diabetes and obesity[27]. One of the aromatic compounds extracted from the stem of *B. gymnorrhiza*, bruguierol C showed moderate activity against gram-positive and gram-negative bacteria including mycobacteria and resistant strains (MICs 12.5 µg/mL)[28].

The lupane caffeoyl ester, 3-(*Z*)-caffeoyllupeol extracted from *B. parviflora* exhibited antimalarial activity with an $EC_{50}$ value of 8.6 µg/mL[8]. Sexangulic acid obtained from *B. sexangula* showed moderate *in vitro* cytotoxicity against human lung cancer (A-549) and human luekaemic (H-L60) cell lines at a concentration of 5 µg/ml[10]. Tagalsin C found in *C. tagal* was found to exhibit moderate cytotoxicity against HeLa human cervical carcinoma cell lines[35]. The dimeric diterpenoid, 8(14)-enyl-pimar-2'(3')-en-4'(18')-en-15'(16')-endolabr-16,15,2',3'-oxoan-16-one and the other terpenoids; tagalsin C, tagalsin I, lup-20(29)-ene-3*β*,28-diol , 3-oxolup-20(29)-en-28-oic acid and 28-hydroxy-lup-20(29)-en-3-one isolated from the roots of the mangrove plant *Ceriops tagal* exhibited antifouling activity against cyprid larvae of the barnacle without significant toxicity[36]. The other nontoxic antifouling compounds identified were ethoxy-*ent*-8(14)-pimarenely-15-one, *ent*-8(14)-pimarene-15*R*,16-diol, stigmasterol and *β*-sitosterol[45]. Tagalsins Q, R and U showed moderate antifeedant activity against the third instar larvae of

**Figure 3-1.** Lupanes from Rhizophoraceae mangroves

*Brontispa longissima* at a concentration of 1 mg/mL[38]. Dolabr-4(17),15(16)-dien-3-one, isopimar-8(14)-en-15,16-diol, isopimar-8(14)-en-16-hydroxy-15-one, lupeol, lup-20(29)-en-3*β*,28-diol and lup-20(29)-en-3*β*-hydroxy-28-oic acid were isolated from the roots of marine mangrove *C. tagal* which were evaluated for the activation of caspase-3 enzyme using caspase-3 colourimetric assay. Caspase-3 enzyme was activated by all compounds in cleaving *p*NA from Ac-DEVD-*p*NA in the presence of caspase-3-inhibitor; Ac-DEVD-CHO[39].

2,6-dimethoxy-*p*-benzoquinone isolated from *R. apiculata* was identified as an active constituent component against fungi, bacteria and boll weevils[55]. Taraxerol and cinnamoyllupeol, are two triterpenoids derived from the leaves and stems of *Rhizophora mangle* L, were found exhibit insecticidal activity towards *Cylas formicarius*: one of the most destructive pests of the sweet potato[51].

Among the compounds isolated from the leaves of *Rhizophora stylosa*, taraxerol has been confirmed to have growth inhibitory effects of Hela and BGC-823 with $IC_{50}$ of 73.4 μmol/L and 73.3 μmol/L, respectively, while *cis*-careaborin may inhibit the growth of BGC-823 and MCF-7 with $IC_{50}$ of 45.9 μmol/L and 116.0 μmol/L, respectively. Furthermore, the presence of astilbin and rutin were initially reported to stimulate the proliferation of mice splenic lymphocytes markedly in a dose-dependent manner[50]. The

**Figure 3-2.** Lupanes from Rhizophoraceae mangroves compounds, (–)-epicatechin, (–)-catechin, 3-O-acetyl-(–)-epicatechin, 3,7-O-acetyl-(–)-epicatechin, (+)-afzelechin, cinchonain 1b and proanthocyanidin B2 were isolated from the same plant displayed DPPH radical scavenging activity which were comparable to that of the positive control butylated hydroxytoluene (BHT). Proanthocyaninidin B2 showed the strongest activity with $IC_{50}$ 4.3 μg/mL, being four fold greater than the positive control, BHT ($IC_{50}$ 18.0 μg/mL). The antioxidant flavan-3-ol glycosides from *R. stylosa* showed an increase in their radical scavenging activities with increase in number of catechol moieties present in the molecules[60].

**3.2 Bioactivities of Mangrove Extracts:** Various publications have reported the biological activities of mangrove extracts. The components of crude alkaloid mixtures from *B. sexangula* and *B. exarista* were identified as tumor inhibitors[6]. A polysaccharide extracted from the leaves of *B. cylindrica*, *R. apiculata* and *R. mucronata* of Rhizophoraceae along with some other mangrove plants exhibited positive activity against human immunodefiency viruses (HIV)[61]. All parts of *Ceriops decandra* have proven antiviral activity[61]. It also possess promising antibacterial[62], antiinflammatory[63], and antidiabetic activity[64]. The leaves and bark extract of *C. tagal* shows antibacterial activity[65]. Phenolics are important components of the leaf extract and hypocotyls of *K. candel* and show excellent antioxidant activities[66,67]. Therefore, *K. candel* can be a good candidate for further development as an antioxidant medicine. During the study on the antibacterial activities of mangrove extracts against two antibiotic resistant

**Figure 4.** Oleananes, ursanes and dammaranes from Rhizophoraceae mangroves

**Triterpene alcohol**

**85**

**Lanostane**

**86**

**Squalene**

**87**

**Fatty acids**

**88**

**89**

**Figure 5.** Triterpene alcohol, lanostane, squalene and fatty acids from Rhizophoraceae mangroves pathogenic bacteria *Staphylococcus aureus* and *Proteus* sp., it was observed that the ethyl acetate extract of *B. Sexangula* and *R. apiculata* also possessed promising antibacterial activity[68]. This antibacterial activity was also reported in a study showing that gallic acid was extracted from hydrolysable tannin from the barks of *R. apiculata*. The gallic acid possessed a significant antiyeast (anticandidal) activity towards some yeast species of medical importance. It is anticipated that gallic acid from *R. apiculata* is a novel antiyeast agent which may be useful in the treatment of candidiasis[69]. Alcoholic extract of the leaves of *Rhizophora apiculata* from the mangrove forest of Sunderbans, West Bengal, India were prepared and displayed manglycemic/anti-hyperglycemic activity in streptozotocin induced diabetic rats fed a glucose bolus. The results of this study revealed that this plant extract had potential hypoglycemic action[70]. The cholinesterase inhibition activity of *R. lamarckii* was established by Natarajan et. al 2009. The antihyperglycaemic effect of *R. mangle* was studied[71]. The leaf extracts of three mangrove plants of Rhizophoraceae family; *Rhizophora mucronata*, *R. apiculata* and *R. annamalayana* were found to have potential anti-diabetic capacity due to the presence of an insulin-like protein[72]. The various studies mentioned, provide scientific support for the use of the mangroves in folklore medicine for the treatment of diabetes.

Various mangrove plants were tested for their antioxidant capacity[65,73]. It was found that the Rhizophoraceae mangroves showed comparatively higher antioxidant capacity which can be attributed to their higher phenolic content. Additionally, the mangrove plants of Rhizophoraceae family are the source of potent antiviral substances[61].

## 4 Chemotaxonomy

The chemical constituents of mangrove plants of the three true mangrove genera (Rhizophoraceae); *Bruguiera*, *Ceriops* and *Rhizophora*, are the diterpenoid class kauranes exist in the genera *Bruguiera* and *Ceriops*, however kauranes are absent in the genus *Rhizophora*. The genus *Bruguiera* is characterised by the presence of disulphides and polydisulphides which are unique to the genus. Thus they can be considered as significant chemotaxonamic markers of this genus. Also, it was observed that *ent*-pimarane coexists with isopimarane in the genus *Bruguiera*. Interestingly, dolabranes only exist in the genus *Ceriops* making it a significant chemotaxonomic marker. Similarly, labdane was found only in the genus *Rhizophora*, making it a significant chemotaxonomic marker of that specific genus.

Furthermore, extensive investigation is needed to identify and classify the chemical constituents of mangrove plants to construct a thorough basis for the chemotaxonamic studies of these versatile plants.

## 5 Conclusions

In this review, the chemistry and bioactivities of mangroves plants of Rhizophoraceae family have been summarised. Two types of diterpenoids; beyerane and pimarane, and three types of triterpenoids; lupane, oleanane and dammarane, are common chemical constituents ubiquitously found in this family, including 268 metabolites. To date, the chemical constituents from all the mangrove plants of this family have not been investigated. It is clear that mangrove plants can provide a new

**Steroids**

**Figure 6.** Steroids from Rhizophoraceae mangroves

**Kauranes**

**Pimaranes**

**Beyeranes**

**Figure 7.** Kauranes, pimaranes and beyeranes (diterpenoids) from Rhizophoraceae mangroves

**Sulphur compounds**

150

151

152

**Figure 8.** Sulphur compounds from Rhizophoraceae mangroves

**Aromatic compounds**

153

**154** R = H
**159** R = OMe

155

**156** $R_1$ = OH, $R_2$ = H, $R_3$ = H
**157** $R_1$ = OH, $R_2$ =OH, $R_3$ = H
**158** $R_1$ = OH, $R_2$ = H, $R_3$ = OH

160

**Figure 9.** Sulphur compounds from Rhizophoraceae mangroves

**Carbohydrate**

161

**Benzoquinone**

162

**Figure 10.** Carbohydrate and benzoquione identified from Rhizophoraceae mangroves

**Phenolic compounds**

163

164

165

**166**

**167**

**168** $R_1$= H, $R_2$ = OH
**169** $R_1$ = OMe, $R_2$ = OH
**170** $R_1$ = OMe, $R_2$ = OMe

**171**

**Figure 11.** Phenolic compounds from Rhizophoraceae mangroves

**Flavonoids**

**173** R = H
**174** R = OH

**175**

**176**

**172** $R_1$ = H, $R_2$ = H, $R_3$ = H
**177** $R_1$ = Ac, $R_2$ = Ac, $R_3$ = Ac
**178** $R_1$ = Ac, $R_2$ = Ac , $R_3$ = H
**179** $R_1$ = Ac, $R_2$ = H, $R_3$ = H

**180** $R_1$ = H, $R_2$ = H
**181** $R_1$ = H, $R_2$ = OH

**183**

**184**

**185**

**186**

**187**

**188**

**189**

**190**

**191**

**192**

**193**

**194**

**195**

**197**

**199**

**Figure 12.** Flavonoids from Rhizophoraceae mangroves

**Lignans**

**214** H-7''/H-8'' = H-7'''/H-8''' = H-7''''/H-8'''' = erythro
**215** H-7''/H-8'' = threo, H-7'''/H-8''' = H-7''''/H-8'''' = erythro

**207**

**208** R = Rhamnosyl

**209**

**210** 7'',8'' = 7''',8''' = erythro
**211** 7'',8'' = erythro, 7''',8''' = threo
**212** 7'',8'' = 7''',8''' = threo

**213**

**Figure 13.** Lignans from Rhizophoraceae mangroves

**Dolabranes**

**216**

**217** $R_1$ = OH, $R_2$ = H, $R_3$ = COOH
**225** $R_1$ = OH, $R_2$= H, $R_3$ = $CH_2$=CH
**231** $R_1$ = H, $R_2$ = H, $R_3$ = $CH_2$=CH
**233** $R_1$ = H, $R_2$ = H, $R_3$ = OH

**227** $R_1$ =H, $R_2$ = H, $R_3$ = $H_2C$=CH
**228** $R_1$ = H, $R_2$ = OH, $R_3$ = $H_2C$=CH
**234** $R_1$ = H, $R_2$ = OH, $R_3$ = COOH
**235** $R_1$ = H, $R_2$ = OH, $R_3$ = HO−$CH_2$C(O)
**236** $R_1$ = H, $R_2$ = OH, $R_3$ = HO−$CH_2$C(O)
**237** $R_1$ = H, $R_2$ = OH, $R_3$ = HO−$CH_2$C(OH)

**218** X = O, $R_1$ = OH, $R_2$ = HO−$CH_2$C(O)
**219** X = O, $R_1$ = OH, $R_2$ = COOH
**220** X = $H_2$, $R_1$ =H, $R_2$ = HO−$CH_2$C(OH)
**229** X = O, $R_1$ = OH, $R_2$ = $H_2C$=CH
**232** X = O, $R_1$ = OH, $R_2$ = OH

**221**

**222**

**223**

**Figure 14.** Dolabranes and dimeric diterpenoids from Rhizophoraceae mangroves

**Figure 15.** Aliphatic alcohols and acids in Rhizophoraceae mangroves

**Figure 16.** Terpenoids, abietane, labdanes, hopanoid, a sesquitepenoid, aromatic and aliphatic ketones in Rhizophoraceae mangroves bank of phytochemical substances that are biologically active substances, with novel structures. It is essential to systematically conserve the biodiversity in the mangrove ecosystem and for the proper of this ecosystem for the future use of humanity.

**Open Access** This article is distributed under the terms of the Creative Commons Attribution License which permits any use, distribution, and reproduction in any medium, provided the original author(s) and source are credited.
